# Brucein D augments the chemosensitivity of gemcitabine in pancreatic cancer via inhibiting the Nrf2 pathway

**DOI:** 10.1186/s13046-022-02270-z

**Published:** 2022-03-10

**Authors:** Juan Zhang, Hong-Xi Xu, William Chi Shing Cho, Wah Cheuk, Yang Li, Qiong-Hui Huang, Wen Yang, Yan-Fang Xian, Zhi-Xiu Lin

**Affiliations:** 1 School of Chinese Medicine, Faculty of Medicine, The Chinese University of Hong Kong, Shatin, N.T. Hong Kong SAR, HK 999077 P.R. China; 2 School of Pharmacy, Shanghai University of Traditional Chinese Medicine, Shanghai, 201203 P.R. China; 3 Department of Clinical Oncology, Queen Elizabeth Hospital, Kowloon, Hong Kong SAR, P.R. China; 4 Department of Pathology, Queen Elizabeth Hospital, Kowloon, Hong Kong SAR, P.R. China; 5 Hong Kong Institute of Integrative Medicine, The Chinese University of Hong Kong, Hong Kong SAR, P. R. China

**Keywords:** Brucein D, Gemcitabine, Chemosensitivity, Pancreatic cancer, Nrf2

## Abstract

**Background:**

Gemcitabine (GEM) is the first-line chemotherapeutic drug used to treat pancreatic ductal adenocarcinoma carcinoma (PDAC), but chemoresistance is often encountered clinically. Nrf2, an oxidative stress responsive transcription factor, is an important contributor to chemoresistance and poor prognosis of PDAC. Brucein D (BD), a naturally occurring quassinoid, has been reported to exert anti-tumor effect in several cancers including PDAC. In this study, we aimed to investigate the efficacy of BD and the role of Nrf2 axes on the chemosensitivity of GEM and elucidate the underlying molecular mechanisms.

**Methods:**

Analyses of clinical samples of PDAC and GEPIA database were first conducted to identify the expression of Nrf2 in PDAC. We then established cell lines with stable deletion of Nrf2 through transfecting lentivirus into PDAC cells. Quantitative real-time PCR (qRT-PCR) and Western blotting were performed to determine the expression of Nrf2 in these cell lines. The effects of BD and Nrf2 axes on PDAC cell proliferation, colony-formation, tumor growth and chemosensitivity were determined both in vitro and in vivo. Orthotopic xenograft and genetically engineered KPC mouse models of PDAC were used to evaluate the anti-pancreatic cancer effects of BD and GEM.

**Results:**

Nrf2 was highly expressed in PDAC in the clinical samples and GEPIA analysis. Gain- and lost-function study demonstrated that Nrf2 affected the chemosensitivity of GEM on PDAC cells both in vitro and in vivo. We further found that BD effectively inhibited PDAC cell proliferation and enhanced the chemosensitivity of GEM. Mechanistic studies revealed that BD sensitized GEM in PDAC cells through the ubiquitin–proteasome-dependent degradation of Nrf2, and downregulating the Nrf2 pathway. Silencing of Nrf2 plus BD treatment resulted in more potent inhibitory effects of GEM. In contrast, Nrf2 activation attenuated the chemosensitivity of GEM, indicating that the action of BD was Nrf2 dependent. Finally, the efficacy of BD alone and in combination with GEM on PDAC was validated on both orthotopic xenograft and genetically engineered KPC mouse models.

**Conclusions:**

BD was able to enhance the chemosensitivity of GEM in PDAC through inhibition of the Nrf2 pathway. Our experimental findings indicate that BD, a potent Nrf2 inhibitor, holds promise for further development into a novel adjuvant therapy for PDAC.

**Supplementary Information:**

The online version contains supplementary material available at 10.1186/s13046-022-02270-z.

## Background

Human pancreatic ductal adenocarcinoma (PDAC) is a highly malignant and lethal digestive tumor. PDAC currently ranks the fourth leading cause of cancer-related death worldwide with a dismal 5-year survival rate, which is less than 5% [1]. Despite the fact that some effective treatments are available, cancer death rates due to PDAC continue to rise unabated. One of the major obstacles in PDAC treatment is the occurrence of resistance to the gemcitabine (GEM)-based chemotherapy [2]. GEM, a cytotoxic nucleoside analog, has been widely used as the standard first-line treatment for advanced PDAC [2, 3], but chemoresistance often occurs clinically. Hence, it is imperative to identify effective adjuvants to enhance the chemosensitivity of GEM in PDAC.

Nuclear factor-erythroid factor 2-related factor 2 (NFE2L2, Nrf2) is an important transcription factor that regulates the antioxidant response by eliciting the expression of genes bearing an antioxidant response element (ARE) in their regulatory regions [4]. The activity of Nrf2 is primarily governed by its physical and functional interaction with the cytosolic repressor Kelch-like ECH-associated protein 1 (Keap1), which facilitates the ubiquitination and subsequent proteasomal degradation of Nrf2 in the cytoplasm via the Cullin 3 ubiquitin ligase complex under normal condition [5]. Following oxidative stress, Nrf2 is released from Keap1, then translocates into the nucleus where it activates ARE-mediated Nrf2 downstream genes like Phase II metabolizing-detoxifying and antioxidant defense enzymes such as NADP(H): quinone oxidoreductase (NQO1), heme oxygenase-1 (HO-1), γ-glutamylcysteine synthetase modifier subunit (γ-GCSm) and Aldo–keto-reductase (AKR1B10) [6–9]. In cancer cells, Nrf2 can also stimulate the multidrug-resistance pretein-1 (MRP1) and multidrug-resistance protein-5 (MRP5) which promote chemoresistance [10–12]. Growing evidence indicates that aberrant Nrf2 signaling is frequently found in multiple cancers including PDAC, and is linked to tumor progression and poor prognosis. Activation of Nrf2 is also correlated with chemotherapy drug resistance in PDAC cells [13–17]. Patients with relatively lower expression level of Nrf2 were found to be more sensitive to chemotherapy [18]. Moreover, Nrf2 deletion in the KPC mice caused a decrease in the formation of precancerous lesions and slowed down the development of invasive PDAC [13, 19]. Therefore, Nrf2 has been considered as a therapeutic target for PDAC prevention and therapy, and the Nrf2 suppression could be exploited for augmentation of efficacy of PDAC therapeutics. In this regard, it is urgently needed to identify agents that could suppress the Nrf2 activity, and to develop them into adjuvant therapy for the GEM-based chemotherapy for PDAC.

Brucein D (BD) is a quassinoid originally isolated from Chinese herb *Bruceae Fructus* (Ya-Dan-Zi in Chinese), which has been used in clinical practice to treat inflammation, malaria, warts and cancers [20]. Previously, we have found BD to have anti-cancer activity in several cancer types such as PDAC, lung cancer and hepatocellular carcinoma via inducing apoptosis, autophagy and oxidative stress through modulating the reactive oxygen species (ROS)/mitogen-activated protein kinase (MAPK) signaling pathway [21–23]. However, data about the combined effects of BD and GEM remains scarce. The present study aimed to explore whether BD could enhance the chemosensitivity of GEM in PDAC, and to elucidate the underlying molecular mechanisms focusing on Nrf2 pathway. By utilizing human PDAC cell lines, we have confirmed that BD efficiently inhibited cell proliferation and enhanced chemosensitivity of GEM in PDAC cells via modulating Nrf2 and its downstream target genes. Using a genetically engineered mouse model of pancreatic cancer, i.e., *Kras*^tm4Tyj^ *Trp53*^tm1Brn^ *Tg* (Pdx1-cre/Esr1*) #Dam/J (KPC tamoxifen-inducible) mouse that can generate PDAC spontaneously, we have verified the anti-PDAC effect of BD in vivo. Our findings unambiguously indicate that BD is a promising adjuvant therapy to augment the chemosensitivity of GEM in PDAC, and the action mechanism involves mitigation of the aberrant Nrf2 expression.

## Methods

### Human tissue specimens

Human PDAC and adjacent normal pancreatic tissue were collected from PDAC patients with informed consent at Queen Elisabeth Hospital, Hong Kong, China. The use of human clinical specimens was approved by the Research Ethics Committee of Kowloon Central / Kowloon East Cluster, Hong Kong, China.

### Cell lines and culture conditions

Human PDAC cell lines PANC-1 and Capan-2 were purchased from the American Type Culture Collection (ATCC). Miapaca-2 cell line was a gift of Prof. XU Hong-xi (Shanghai University of Traditional Chinese Medicine, Shanghai, China). All of the cell lines were cultured in Dulbecco’s Modified Eagle medium (DMEM) supplied with 10% fetal bovine serum (FBS) and 10 U/mL penicillin–streptomycin in an incubator at 37 °C and 5% CO_2_.

### Gene expression correlation with stage and survival analysis

The correlation between gene expression and clinical stage was determined using Gene Expression Profiling Integrative Analysis (GEPIA, http://gepia.cancer-pku.cn) [24]. The correlation between gene expression and overall survival (OS) was established using the Cox model. Nrf2 with higher expression in the PDAC samples had corresponding lower survival.

### Fluorescence-activated cell sorting (FACS) analysis

For the flow cytometry experiment, Annexin V/PI staining kit (BD Pharmingen™, USA) was used to detect the cell apoptosis following the manufacturer’s instruction. Apoptotic cells were counted by a Cytomics™ FC500 flow cytometer (Beckman Coulter, Fullerton, CA, USA). Intracellular ROS concentration was measured by Total ROS Assay Kit (Invitrogen, Carlsbad, CA, USA) according to the manufacturer’s manual. All the data generated by flow cytometer were analyzed using the Kaluza software (Beckman Coulter).

### Western blot analysis

Proteins were extracted from cell lysates or tumor tissues with lysis buffer, supplemented with a complete protease inhibitor cocktail (Thermo Scientific, USA). The concentrations of the protein extracts were determined by bicinchoninic acid (BCA) test. Protein samples were resolved by SDS-PAGE and transferred to a polyvinylidene difluoride membrane. After blocking nonspecific binding with TBS/T (0.1%) containing 5% non-fat milk for 1 h at room temperature (RT), the membranes were then incubated overnight at 4 °C with primary antibody diluted in 3% BSA in TBS/T (0.1%). The membrane was washed with TBS/T four times to remove the unbound antibody and then incubated with the secondary antibody (HRP-conjugated goat anti-mouse IgG or goat anti-rabbit IgG, 1: 2,500; Cell Signaling Technology) for 1 h at room temperature. Protein bands were visualized with an ECL kit (Invitrogen, Carlsbad, CA, USA). All antibodies and their dilutions used in this experiment were listed in the Additional file 1, Table S1.

### Immunofluorescent staining

PDAC cells were plated on coverslips and allowed to adhere overnight and expose to BD (2.5 µM) for 24 h. Then the cells were fixed with 4% paraformaldehyde/PBS, blocked with 5% BSA in PBS and incubated with Nrf2 primary antibody (1:100; Santa Cruz #sc-365949) in PBS containing 3% BSA, followed by Cy3-labeled secondary antibody (Abcam, United Kingdom). Nuclei were stained with DAPI (Santa Cruz, Texas, USA). Images were visualized using an inverted fluorescent microscope (Carl Zeiss, Germany).

### Lentivirus transfection of Nrf2 shRNA and stable cell lines

Target cells (Miapaca-2, Capan-2, PANC-1) were plated in 24-well plates at the density of 2 × 10^4^ cells/well with complete growth medium, and then incubated overnight to improve the adherence of cells to the plates. For lentivirus transduction, the cells were infected with either Nrf2 shRNA or control shRNA lentiviral particles (Santa Cruz, sc-37030-V; sc-108080) in serum-free growth medium with 5 μg/mL polybrene at multiplicities of infection (MOI) of 20, 20, 4 separately. The plates were gently shaken and incubated overnight. Then the medium was replaced by fresh DMEM complete medium, and the cells were incubated for additional 48 h to allow the shRNA to reach its maximum effect. After lentivirus transduction, cells were reseeded in 6-well plates and treated with antibiotics puromycin to select stable clones expressing the shRNA. After the first dose, the medium was replaced with the fresh medium containing puromycin (1, 2 and 5 μg/mL) every 2–3 days until resistant colonies can be identified. Stable cell lines were verified by qRT-PCR and western blot.

### Measurement of Nrf2 protein stability and ubiquitination

Cells were treated with BD (1.5 µM) and with MG132 (20 µM). After treatment for a given time, the cells were harvested for the western blot analysis of the Nrf2 protein level. The half-time of Nrf2 in cells was detected by cycloheximide (CHX)-chase analysis. Cells were co-treated with or without BD (1.5 µM) with CHX (25 µM). Total cell extracts were prepared at indicated time points following treatment with CHX, and then cell extracts were detected by western blot. For analysis of Nrf2 ubiquitination, cells were treated with or without BD (1.5 µM) for the indicated time periods (0–48 h), and the level of ubiquitination was detected by western blot using an anti-ubiquitin antibody.

### Molecular docking of BD on human Keap-1 and Nrf2 complex

SwissDock (URL: www.swissdock.ch) was used to perform the molecular docking analysis of BD on Keap-1: Nrf2 complex. The 3D structure of BD was downloaded from ZINC webpage with the number ZINC8221322 (UCSF; San Francisco, CA, USA). Crystal structure of Keap-1: Nrf2 (PDB ID:2FLU) was downloaded from RCSB PDB Bank (http://www.pdb.org). The docking results were analyzed using UCSF Chimera 1.11.1 (RVBI, UCSF; San Francisco, CA, USA). Ligand binding results with negative △G values were regarded as having an affinity in the binding between BD and Keap-1: Nrf2. The number of possible hydrogen bonds and the bond lengths were determined by the Find H-Bond tool in UCSF Chimera. All docking procedures were performed using Windows 10.

### PDAC mouse models

Six-week-old female BALB/c nude mice were obtained from the Laboratory Animal Services Centre, The Chinese University of Hong Kong (CUHK). Genetically engineered transgenic mouse model (GEMM), which has the mixed background Kras^tm4Tyj^ Trp53^tm1Brn^ Tg (Pdx1-cre/Esr1*) #Dam/J (KPC tamoxifen-inducible), was purchased from the Jackson laboratory (Stock number: 032429) (Bar Harbor, Maine, USA). PCR was applied for the genotyping of the transgenic mice (genotyped for the presence of KRAS, P53, and Cre). The primer sequences used for the genotyping of transgenic mice were presented in the Additional file 1: Table S2. All animals were kept in a pathogen-free environment with free access to food and water. All animal experiments were conducted according to the ethical policies and procedures approved by the Animal Experimentation Ethics Committee of CUHK (Ref. No.: 19/079/NSF and 20/169/MIS).

For the orthotopic implantation, Capan-2, control non-specific shRNA (shControl) or Nrf2-targeting shRNA (shNrf2)-transfected Miapaca-2 cells (1.5 × 10^6^ cells/100 μL) were injected into the pancreatic tail of each nude mouse. The mice were randomly divided into four groups (8 mice per group) 1 week after tumor implantation (day 1): Vehicle control (0.5% DMSO, daily, i.g) group, BD (2 mg/kg, daily, i.g.) group, GEM (20 mg/kg twice a week, i.g.) group, and BD (2 mg/kg, daily, i.g.) plus GEM (20 mg/kg twice a week, i.g.) group. At the end of drug treatment, the mice were sacrificed and the tumor tissues harvested for western blot and immunohistochemistry analysis. Tumor volumes were measured using a vernier caliper and calculated using the following formula: [(shortest diameter)^2^ × (longest diameter) / 2].

For the GEMM of PDAC, the KPC mice were received with the intraperitoneal injection of tamoxifen (1.5 mg/mouse) for 5 consecutive days at 4 weeks of age to induce metastatic pancreatic carcinoma. Tamoxifen was diluted in corn oil (Sigma-Aldrich) to afford the injection solution. The KPC mice were randomly divided into 4 groups (7 mice per group): Vehicle control group (0.5% DMSO, i.g.), BD (2 mg/kg, once daily, i.g.) group, GEM (20 mg/kg twice weekly, i.g.) group, BD (2 mg/kg, once daily, i.g.) plus GEM (20 mg/kg twice a weekly, i.g.) group. BD, GEM or vehicle were given to the KPC mice for 4 consecutive weeks. At the end of drug treatment, blood samples (500 µL) were collected from the mice under anaesthesia, and then the mice were sacrificed by cervical dislocation. Serum was collected after centrifugation at 2,000 xg for 15 min in a refrigerated centrifuge. The liver and renal toxicities of BD were determined by measuring the levels of aspartate aminotransferase (AST), alanine aminotransferase (ALT) and creatinine using respective activity assay kits (Nanjing Jiancheng Bioengineering Institute, China).

### Histopathological and immunohistochemical analysis

Tissues for histopathological analysis were fixed in 4% paraformaldehyde (PFA) at 4 °C overnight before paraffin embedding. All sections used for histological analysis were cut into 5 μm thickness using a microtome. Histological characterization and consequent scoring of neoplastic lesion on hematoxylin and eosin (H&E)-stained sections of pancreas were performed. A registered pathologist provided supervision and confirmation for this work. Masson’s trichrome staining was performed to determine the collagen deposition using the Trichrome stain (Masson) Kit (HT15-1KT, Sigma-Aldrich, Heatherhouse, United Kingdom) according to the manufacturer’s instructions. The percentages of the stained area were calculated using Image J software.

For immunohistochemical analysis, paraffin sections were de-waxed with xylene, and re-hydrated with gradient ethanol before they were stained with antibodies against Ki-67 (1:100), Nrf2 (1:100) and NQO1 (1:100). All sections were photographed using inverted fluorescence microscope (Carl Zeiss, Germany). The proportion of IHC-positive cells was determined in the randomly selected microscopic fields.

### Statistical analysis

Data were presented as the mean ± SD. Student’s *t*-test was used for comparison between two different groups, and one-way analysis of variance (ANOVA) was used for multiple comparisons. A two-tailed value of *p* < 0.05 was considered statistically significant. Statistical analyses were carried out using GraphPad Prism 8.0 (Version 8, GraphPad Software, Inc., CA, USA).

The detailed methodology of qRT-PCR and functional experiments can be found in the Supplementary Materials.

## Results

### Up-regulation of Nrf2 in human PDAC and predicts poor prognosis

**Fig. 1:**
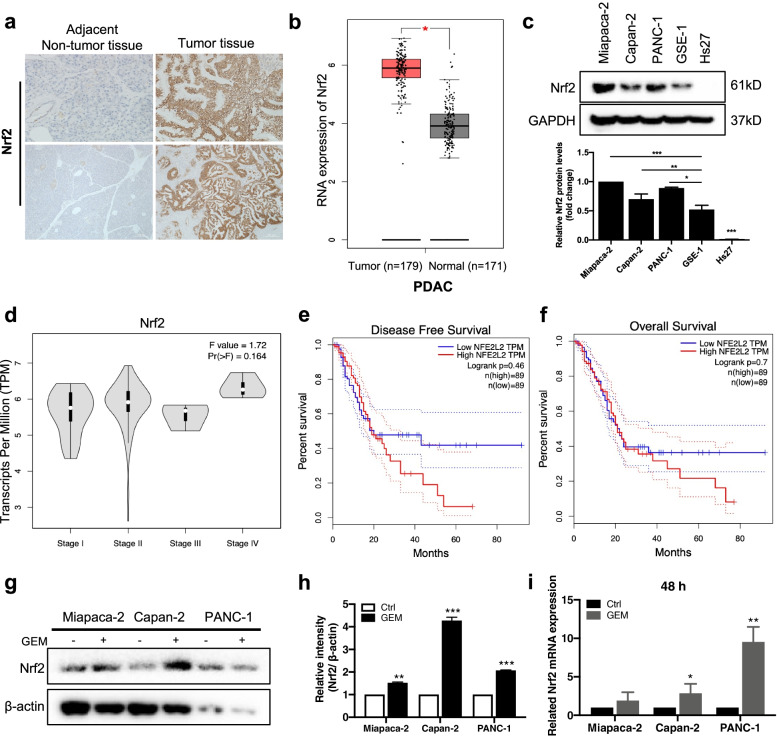
Up-regulation of Nrf2 in human PDAC and predicts poor prognosis. **a** The paired tumor and adjacent normal tissues in pancreatic cancer patients were stained with IHC (Scale bar: 50 & 200 μm) for detection of Nrf2 protein levels (*n* = 4). **b** The expression level of NFE2L2 (Nrf2) in PDAC (*n* = 179) and normal pancreas (*n* = 171) was analyzed using GEPIA. **c** Nrf2 expression in Miapaca-2, Capan-2, PANC-1, GSE-1 and Hs27 cells were detected by western blot. **d** Expression of Nrf2 in different clinical stages of PDAC patients. **e–f** Kaplan–Meier curve showed the correlation between Nrf2 expression and disease-free survival, overall survival of PDAC patients analyzed using GEPIA. **j-h** Immunoblotting for Nrf2 expression in GEM-treated Miapaca-2, Capan-2 and PANC-1 cells. β-actin was used as a loading control. **i** The mRNA expression of Nrf2 in Miapaca-2, Capan-2, and PANC-1 cells. The cells were treated with GEM for 48 h. Data were presented as the mean ± SD. *p < 0.05, **p < 0.01 and ***p < 0.001

To explore the clinical significance of Nrf2, we determined Nrf2 expression in PDAC patients. The Nrf2 expression was markedly higher in the PDAC tissues as compared to the adjacent noncancerous tissues (*n* = 4) (Fig. 1a & Supplementary Fig. S1a). H&E analysis of the tumors also showed a reduction in cell density in adjacent normal tissue than in the cancer tissue (Supplementary Fig. S1b). A large-scale dataset analysis by using GEPIA (http://gepia.cancer-pku.cn) and Oncomine database confirmed that Nrf2 was overexpressed in the PDAC tissues when compared to that in the normal tissues (Fig. 1b & Supplementary Fig. S1c). In addition, the protein levels of Nrf2 in human PDAC cells (Miapaca-2, Capan-2, PANC-1) and normal human gastric epithelial cells (GSE-1) and human fibroblast cells (Hs27) were detected using western blot, and it was found that Nrf2 protein levels in the cancer cells were significantly higher than those in the normal cells (Fig. 1c). It also found that the patients whose tumors were clinically staged later had higher expression levels of Nrf2 (Fig. 1d). Moreover, Kaplan–Meier survival analysis indicated that high expression of Nrf2 in PDAC tissues was associated with a shorter overall survival of PDAC patients (Fig. 1e-f). We also detected the influence of GEM on the expression of Nrf2 and found that GEM treatment upregulated the Nrf2 expression in all PDAC cell lines at the protein and mRNA levels (Fig. 1g-i & Supplementary Fig.S1d). Taken together, these data suggest that there is a positive role of Nrf2 in PDAC progression, and unfortunately, GEM treatment resulted in the activation of Nrf2 signaling pathway.

### BD potentiates the sensitivity of PDAC cells to GEM

**Fig. 2:**
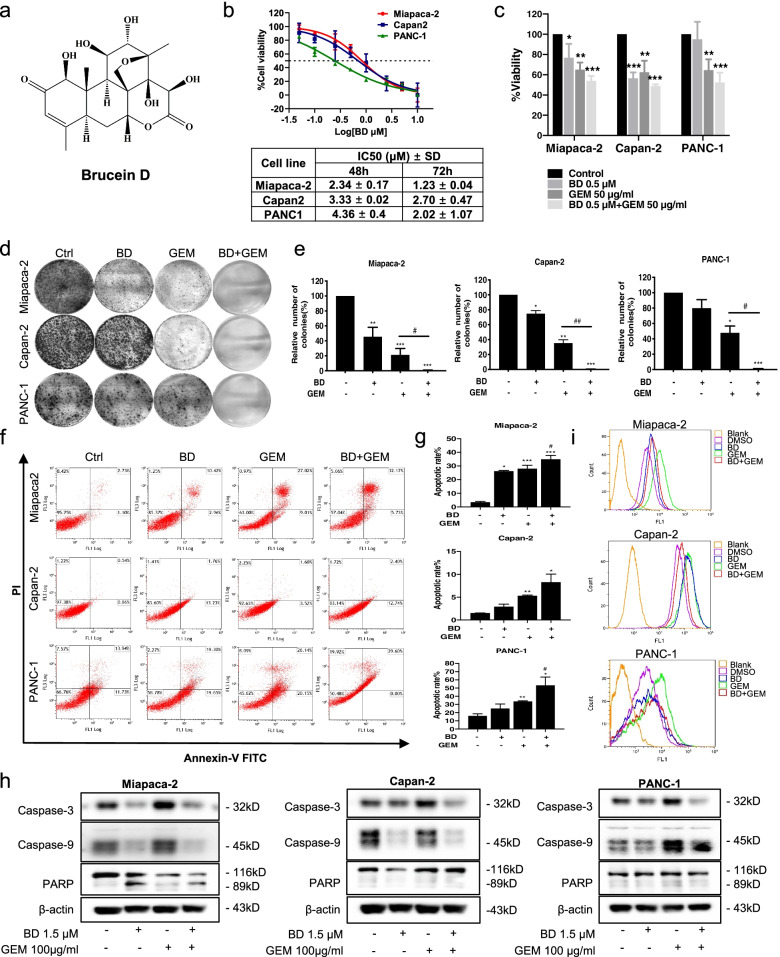
BD enhances the sensitivity of GEM in human PDAC cells. **a** The chemical structure of BD. **b** Miapaca-2, Capan-2 and PANC-1 cells were treated with different concentrations of BD for 48 or 72 h, and the cell viability was assessed using MTT assay. **c** Pancreatic cell lines treated with BD (0.5 µM) or/and GEM (50 µg/mL) for 48 h, and MTT assay was used to measure the cell viability. **d-e** Colony formation assay. **f-g** Flow cytometric analysis for cellular apoptosis. **h** The expression level of Caspase-3, caspase-9 and PARP subjected to BD and GEM treatment in Miapaca-2, Capan-2 and PANC-1 cells were detected by western blot. **i** Flow cytometry analysis of ROS levels in PDAC cells treated with BD or GEM and their combination. Data were presented as the mean ± SD. *p < 0.05, **p < 0.01 and ***p < 0.001 compared with the control group. ^#^*p* < 0.05, ^##^*p* < 0.01 compared with the GEM alone treatment group

Mobility assay was performed to evaluate the effect of BD in the PDAC cell lines (The chemical structure of BD is shown in Fig. 2a). BD significantly inhibited the proliferation of Miapaca-2, Capan-2 and PANC-1 cells with similar IC_50_ values (Fig. 2b). Since BD possesses potent inhibitory effect on PDAC cell proliferation, we speculated that BD could have a synergetic effect with GEM in PDAC in vitro. The MTT assay showed that BD effectively sensitized the Miapaca-2, Capan-2 and PANC-1 cells to GEM treatment (Fig. 2c). In addition, the combination of BD and GEM exerted synergetic inhibitory effect on the colony formation of PDAC cells (Fig. 2d-e), and increased the number of cells undergoing apoptosis, when compared with GEM treatment alone, through activating the caspase-3, caspase-9 and PARP protein levels in PDAC cells (Fig. 2f-h). BD also augmented the GEM effect on the ROS accumulation following 24 h treatment (Fig. 2i). These results unequivocally indicated that BD could enhance the chemosensitivity of GEM and accentuate the effect of GEM on the ROS accumulation in Miapaca-2, Capan-2 and PANC-1 cells, thereby leading to augmented cellular apoptosis.

### BD enhances the anti-tumor activity of GEM in KPC mouse model

**Fig. 3:**
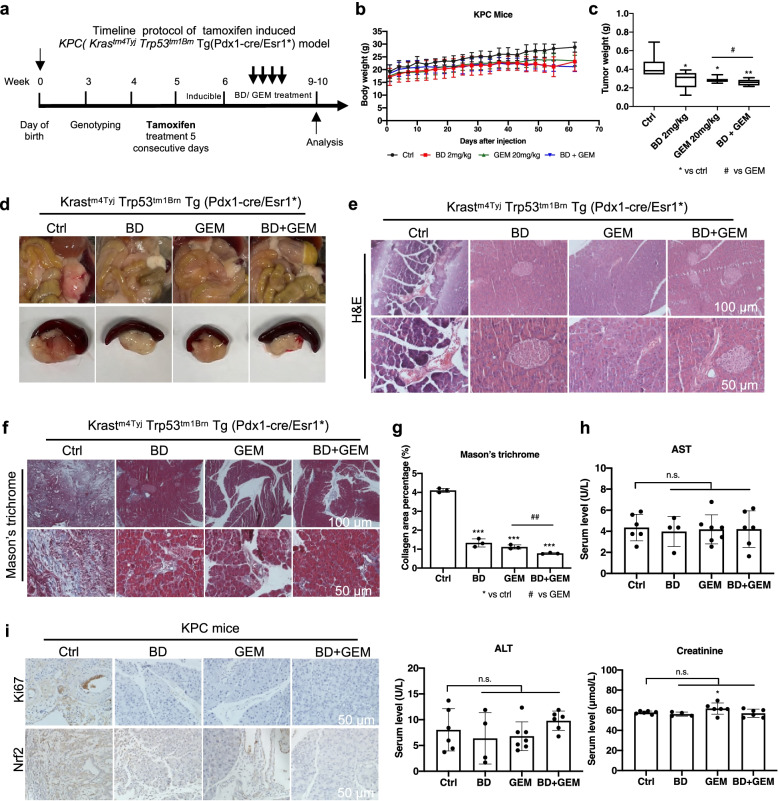
BD enhances the anti-tumor activity of GEM in KPC mouse model. **a** The timeline of experiments on KPC model. **b** Body weight of the mice was measured every other day. **c-d** Representative macroscopic images and tumor weight of PDAC in KPC mice treated with BD, GEM and their combination. **e–f** Histopathological analysis of the tumors of KPC mice and representative images of Masson’s trichrome staining. **g** Quantification of Masson’s trichrome staining in the tumor tissue of KPC mice. **h** Serum levels of AST, ALT and creatinine of KPC mice (*n* = 4 to 7 per group). **i** Paraffin-embedded KPC tumor tissues were stained with IHC for evaluation of Ki-67 and Nrf2 expression (Scale bar: 50 μm). Data were presented as the mean ± SD. *p < 0.05, **p < 0.01 and ***p < 0.001 compared with the control group; n.s., no significant. ^#^*p* < 0.05 and ^##^*p* < 0.01 compared with the GEM alone treatment group

To further corroborate the therapeutic effect of BD in PDAC, we assessed whether BD could also sensitize the anti-PDAC effect of GEM in KPC transgenic mouse model. Six-week-old KPC mice were treated with vehicle, BD (daily), GEM (twice a week) and their combination at indicated time (Fig. 3a). As expected, treatment with the combination of BD and GEM exerted more potent anti-PDAC effect in KPC mice when compared with GEM treatment alone. Accordingly, KPC mice treated with BD had a decreased tumor weight (Fig. 3b-d) when compared to the vehicle-treated control. We further evaluated the anti-fibrotic role of BD in KPC mice using Masson’s trichrome staining. The Masson’s trichrome-stained area was significantly reduced following BD, GEM or their combination treatment (Fig. 3e-g). Furthermore, we also found that BD, GEM or their combination treatment markedly down-regulated the protein levels of Nrf2 and Ki67 in the tumor tissues of KPC mice (Fig. 3i).

We also evaluated the possible chronic liver and renal toxicities of BD treatment using blood biochemical analysis. At the end of BD, GEM or their combination treatment, blood samples were collected, and the serum AST and ALT levels measured to determine the liver toxicity of the KPC mice. As shown in Fig. 3h, the AST and ALT levels in BD, GEM and their combination group were similar to those of the control group. Likewise, we evaluated the renal toxicity of BD, GEM and their combination by measuring the creatinine level. No overt renal toxicity was observed among these groups. Moreover, no abnormality was seen in the liver and kidney tissues as examined histopathologically (Supplementary Fig. S2a-b). Furthermore, BD induced no treatment-related abnormality concerning the gross anatomy and histological morphology (Supplementary Fig. S2c). Collectively, these data suggested that BD reduced the tumor burden and enhanced the chemotherapeutic effect of GEM in KPC mice, while it exerted no overt systemic toxicity.

### BD selectively inhibits the Nrf2 pathway

**Fig. 4:**
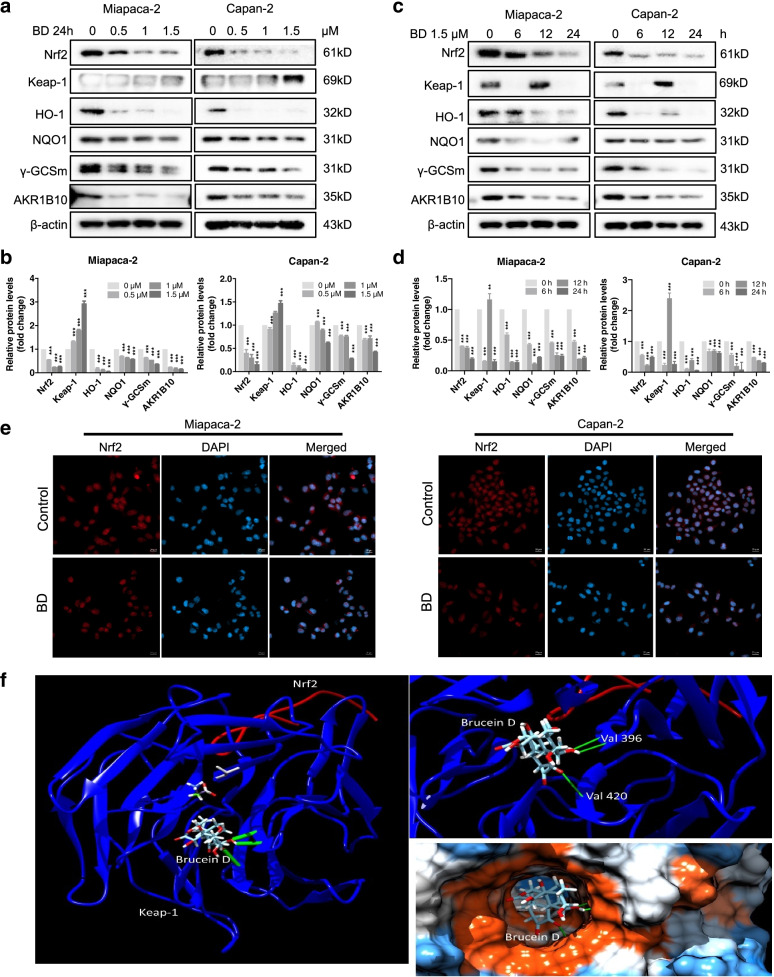
BD selectively inhibits the Nrf2 pathway. **a-b** Effects of BD on the protein level of Nrf2 in PDAC cells. Miapaca-2 and Capan-2 cells were treated with BD (0–1.5 µM) for 24 h. **c-d** BD (1.5 µM) treatment for the indicated time periods (0–24 h). **e** Immunofluorescence staining of endogenous Nrf2 in Miapaca-2 and Capan-2 cells treated with or without BD (1.5 µM) for 24 h. All images were shown at 20 µm. (**f**) BD docking with Keap-1 and Nrf2. The blue ribbon represents the Keap-1 amino acid chain, the red ribbon represents the Nrf2 amino acid, the green bonds represent the hydrogen bond. Val is the abbreviation of valine

As up-regulation of Nrf2 expression was observed in PDAC and the GEM-treated cells, and BD could enhance the sensitivity of PDAC cells to GEM, we then evaluated the inhibitory effect of BD on the protein level of Nrf2. The expression level of Nrf2 was significantly attenuated in Miapaca-2 and Capan-2 cells after treatment with BD (0, 0.5, 1, 1.5 μM) (Fig. 4a-b). An inhibitory effect of BD (1.5 µM) on Nrf2 protein level in PDAC cells was also observed. It should be noted that the protein level of Nrf2 began to decrease from 6 h onward after BD treatment (Fig. 4c-d). In addition to Nrf2, the protein levels of Nrf2-target genes, including Keap1, HO-1, NQO1, AKR1B10 and γGCSm, were also down-regulated in a dose and time-dependent manners. The above experimental results amply suggested that BD could effectively inhibit Nrf2 signaling in PDAC cells.

To elucidate the possible mechanism underlying the inhibitory effect of BD on Nrf2 signaling pathway, immunofluorescence staining was applied to observe the translocation of Nrf2 in PDAC cells. As shown in Fig. 4e, the dissociation from the membrane into the cytoplasm was not found after BD treatment. Moreover, an endogenous Nrf2 immunostaining assay further corroborated the inhibitory effect of BD on the protein level of Nrf2.

We also assessed the relationship between BD and Nrf2 using molecular docking. The results revealed that BD could bind to Keap-1:Nrf2 (2flu) at Keap-1 amino acid chain to form 3 hydrogen bonds at valine 369 (O–H, 2.394 Å, and HN-O, 2.079 Å) and valine 420 (HN-O 2.537 Å), respectively, with the binding energy of -8.18 kcal/mol (Fig. 4f). Based on the above experimental and molecular docking results, we conclude that BD is a potent Nrf2 inhibitor. Interestingly, we also found that brusatol (also a quassinoid compound), a known Nrf2 inhibitor, also bound to Keap-1:Nrf2 (2flu) in a manner similar to that of BD (Supplementary Fig. S3a).

### Knockdown of Nrf2 enhances the chemosensitivity of GEM in PDAC

**Fig. 5:**
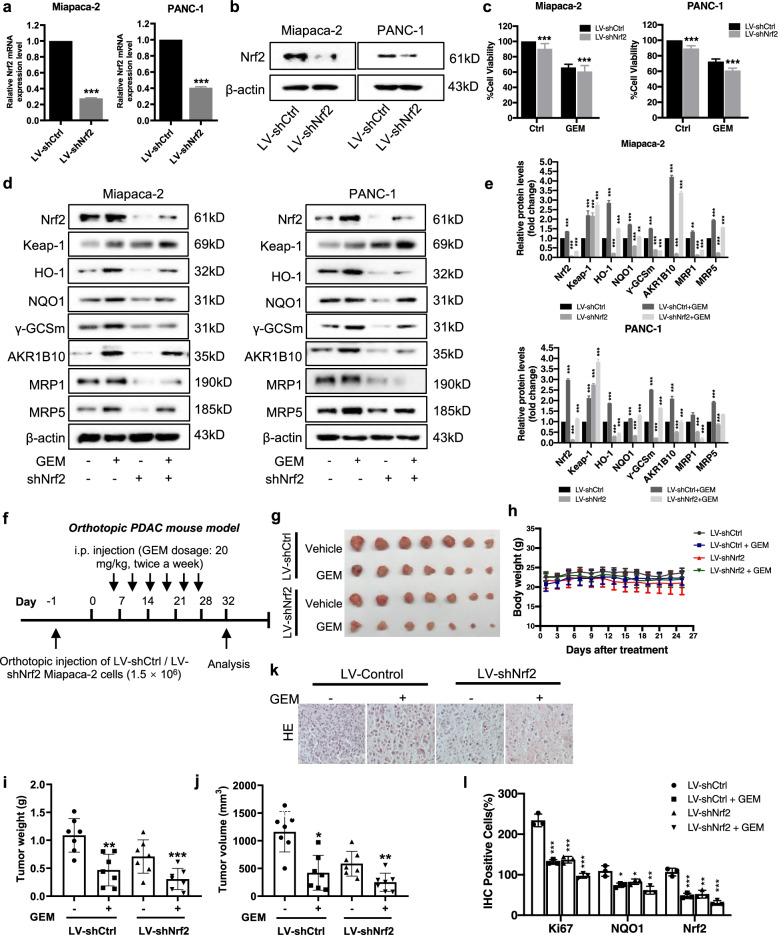
Nrf2 knockdown enhances the chemosensitivity of GEM in human PDAC cells. **a-b** The Nrf2 mRNA and protein were stably knocked down in Miapaca-2 and PANC-1 cells. LV-shNrf2, recombinant lentivirus deletion Nrf2; LV-shCtrl, recombinant lentivirus negative control. **c** Effects of GEM treatment on the viability in the Nrf2-silenced and non-silenced cells. **d-e** Effects of GEM treatment on the protein levels of Nrf2, Keap1, HO-1, NQO1, γGCSm, AKR1B10, MRP1 and MRP5 in the Nrf2-knockdown Miapaca-2, Capan-2 and PANC-1 cells. **f** Schematic drawing of the experimental design on the orthotopic PDAC mouse model. **g** Representative images of tumor and the quantification of tumor weight (**i**) and tumor volume (**j**) (*n* = 7). **h** Body weight of the animals were measured every three days followed by GEM treatment. **k-l** Paraffin-embedded orthotopic tumor tissues were sectioned and stained with H&E (Scale bar: 20 μm), and quantification of IHC for evaluation of Ki-67, Nrf2 and NQO1 expression. Data were presented as the mean ± SD. *p < 0.05, **p < 0.01 and ***p < 0.001 compared with the control group

Since Nrf2 is over-expressed in cancer tissues, and Nrf2 over-expression is known to be associated with chemoresistance. A reduction in Nrf2 expression should help sensitize cancer cells to chemotherapeutic drugs. We hypothesized that deletion of Nrf2 could lead to the chemosensitivity of GEM in PDAC. To verify this hypothesis, we ablated Nrf2 in Miapaca-2 and PANC-1 cells using transfection with a Nrf2-lentivirus construct. As shown in Fig. 5a-b, a pool of Nrf2 shRNA exhibited strong Nrf2 elimination efficacies in both the mRNA expression and protein levels. We then tested the sensitivity of GEM in Miapaca-2 LV-shNrf2 and PANC-1 LV-shNrf2 cells. As predicated, the Miapaca-2 and PANC-1 cells with Nrf2 knockdown were more sensitive to GEM than the LV-shCtrl cells (Fig. 5c). In addition, upon GEM treatment, the protein levels of Nrf2, Keap1, HO-1, NQO1, γGCSm, AKR1B10, MRP1 and MRP5 were markedly decreased in Miapaca-2 and PANC-1 LV-shNrf2 cells when compared with cells transfected with empty vector (Fig. 5d-e). Similar results were also obtained in Capan-2 cells, which were shown in Figure S4a-e. These results strongly indicate that Nrf2 contributes substantially to PDAC progress, and the deletion of Nrf2 successfully sensitizes PDAC cells to GEM.

### Nrf2 deletion suppresses the orthotopic pancreatic tumor growth and improves the chemosensitivity of GEM in vivo

Moreover, similar results that Nrf2 knockdown enhances the sensitivity of GEM were also obtained in vivo. Miapaca-2 LV-shCtrl cells and Miapaca-2 LV-shNrf2 cells were directly injected into the tail of the pancreas of the nude mice to establish an orthotopic model of pancreatic cancer, followed by treatment with or without GEM (Fig. 5f-j). As shown in Fig. 5g, Miapaca-2 LV-shNrf2 group showed an inhibitory effect on tumor as compared with LV-shCtrl group, while treatment with GEM presented better inhibitory effects on tumor growth than Miapaca-2 LV-shCtrl group. No obvious changes in the body weight were found in the animals (Fig. 5h). In addition, LV-shNrf2 group had lower protein levels of Nrf2, HO-1, NQO1, AKR1B10, γGCSm, MRP1 and MRP5 when compared with LV-shCtrl (Supplementary Fig. S4f-g). The H&E staining of excised tumors showed a significantly reduction in cell density in GEM treated LV-shNrf2 group compared with LV-shCtrl group (Fig. 5k). Furthermore, IHC analysis revealed that knockdown of Nrf2 markedly reduced the positive cells of the proliferation marker of Ki-67, and NQO1 and Nrf2 (Fig. 5l & Supplementary Fig. S4h). These results were indicative that knockdown of Nrf2 could inhibit the orthotopic pancreatic tumor growth by improving the sensitivity of GEM to PDAC in vivo.

### Nrf2 activation promotes the chemoresistance of GEM

**Fig. 6:**
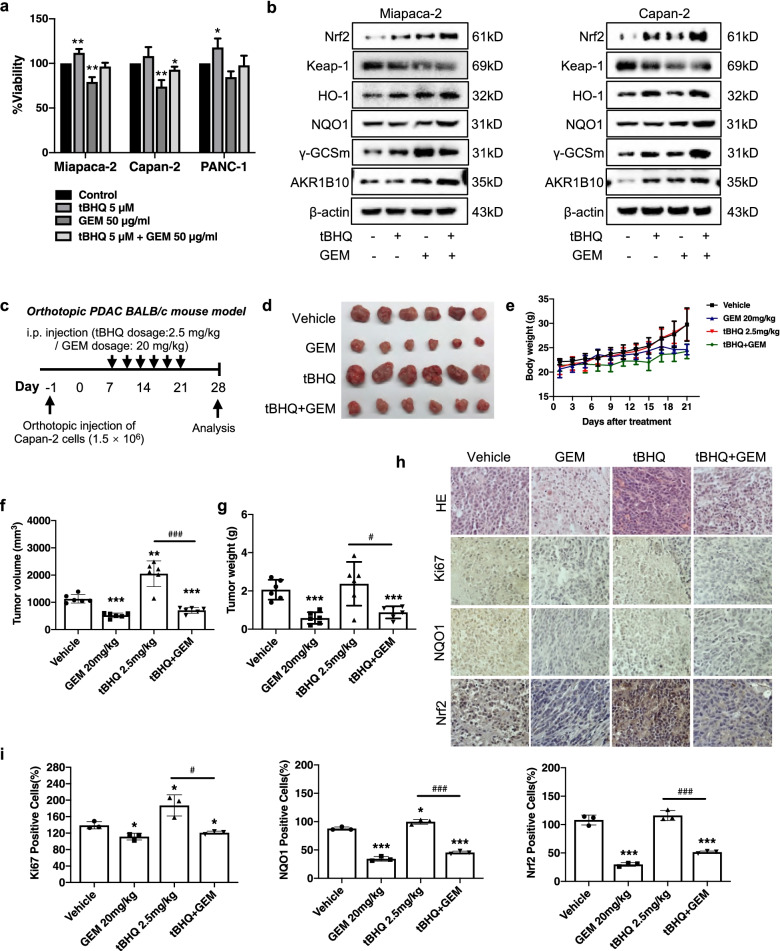
Accentuation of Nrf2 augments the chemoresistance of GEM. **a** The cell viability was determined using MTT assay after treatment with tBHQ, GEM or their combination for 48 h. **b** Effects of Nrf2 activation on protein levels of HO-1, NQO1, γGCSm and AKR1B10 in Miapaca-2 and Capan-2 cells following tBHQ, GEM or their combination treatment. **c** Schematic design of the experiments on the orthotopic PDAC mouse model. **d** Representative tumor images, and the tumor volume (**f**) and tumor weight (**g**). **e** The body weight of each group of the animals (*n* = 6). (h-i). Representative images of H&E and IHC staining of Nrf2, Ki-67 and NQO1 in the tumor tissues (Scale bar: 20 μm). Data were presented as the mean ± SD. *p < 0.05, **p < 0.01 and ***p < 0.001 compared with the control group. ^#^*p* < 0.05, ^###^*p* < 0.001 compared with the GEM alone treatment group

To further verify the effect of Nrf2 on the chemosensitivity of GEM, we pharmacologically activated Nrf2 using Nrf2 inducer tert-butylhydroquinone (tBHQ) in vitro and in vivo. As shown in Fig. 6a, treatment with tBHQ increased the cell viability and enhanced the chemoresistance of GEM in all PDAC cell lines. Immunoblotting analysis unraveled that over-expression of Nrf2 conspicuously activated Nrf2 and Nrf2-targeted gene expression (Fig. 6b). Additionally, we also established an orthotopic mouse model using Capan-2 cells (Fig. 6c). In this model, intraperitoneal injection of tBHQ (2.5 mg/kg) significantly increased the tumor volume and tumor weight, as compared with the vehicle control group and the GEM treatment group (Fig. 6f-g). Moreover, tBHQ treatment also suppressed the anti-tumor effect of GEM, as compared with the GEM alone, leading to larger volume and weight of the xenograft tumor (Fig. 6d). The increases in the body weight seen in the tBHQ and control mice might be the result of more rapid tumor growth in the tBHQ and control group (Fig. 6e). In addition, immunohistochemical analysis revealed that tBHQ-induced activation of Nrf2 significantly accentuated the numbers of Ki-67, Nrf2 and NQO1 positive cells (Fig. 6h-i). These results amply demonstrated that activation of Nrf2 promoted the chemoresistance on GEM, thereby promoting the tumor growth in the Capan-2 cells-derived orthotopic xenograft.

### BD promotes the ubiquitin–proteasome dependent degradation of Nrf2

**Fig. 7:**
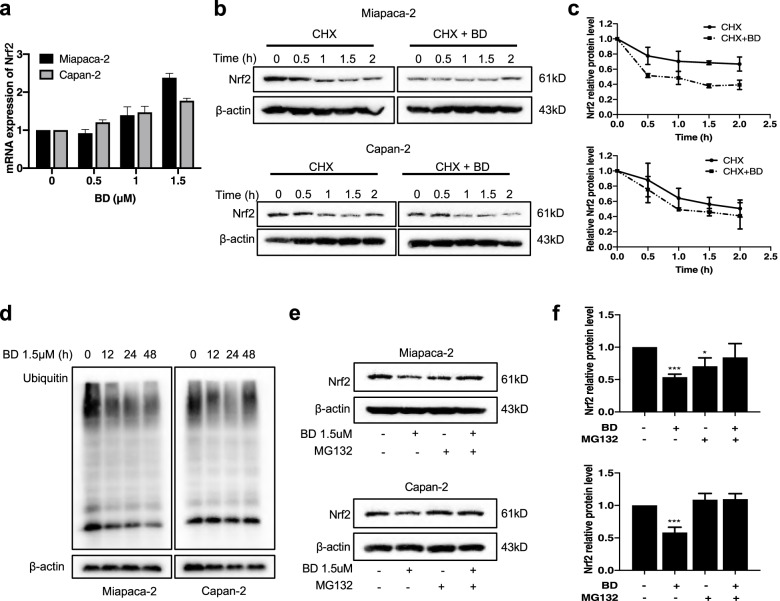
BD promotes the ubiquitin–proteasome dependent degradation of Nrf2. **a** The relative Nrf2 mRNA levels as analyzed by qRT-PCR. **b-c** Miapaca-2 and Capan-2 cells were treated with 25 μM CHX with or without BD (1.5 μM) for the indicated time and the cell lysates were analyzed by immunoblotting. **d** Miapaca-2 and Capan-2 cells treated with BD (1.5 μM) for the indicated time periods (0–48 h), and the level of ubiquitin was detected by immunoblotting using an anti-ubiquitin antibody. **e–f** Miapaca-2 and Capan-2 cells were treated with BD (1.5 µM), MG132 (20 µM), or the combination of BD and MG132 for 8 h, and the protein level of Nrf2 were detected by immunoblotting. Data were presented as the mean ± SD. *p < 0.05, **p < 0.01 and ***p < 0.001 compared with the control group

As BD sensitizes the PDAC cells to GEM through the downregulation of Nrf2 expression, we further explored the mechanism on how BD regulates Nrf2 in PDAC cells. First, the effect of BD on the transcription of Nrf2 was determined. RT-PCR results showed that Nrf2 mRNA level remained unchanged after BD treatment in the two cell lines (Fig. 7a), suggesting that BD did not affect the transcription or mRNA stability of Nrf2. In the presence of BD, the half-life of the Nrf2 protein was reduced in both Miapaca-2 and Capan-2 cells, as measured by cycloheximide (CHX) chase assays (Fig. 7b-c), indicating the accelerated degradation of the Nrf2 protein. We next evaluated the effect of BD on the Nrf2 ubiquitination level using western blotting, and the results showed that the ubiquitination level was accentuated upon BD treatment in PDAC cells (Fig. 7d). Furthermore, we employed MG-132 to inhibit 26 s proteasome activity in Miapaca-2 cells. We found that the BD-induced down-regulation of Nrf2 was significantly attenuated. Similar results were observed in Capan-2 cells (Fig. 7e-f). The above experimental results conspicuously indicate that BD inhibits Nrf2 through promoting its ubiquitin–proteasome degradation rather than decreasing the synthesis of the Nrf2 protein.

### Nrf2 knockdown plus BD enhances the chemosensitivity of GEM in human PDAC cells

**Fig. 8:**
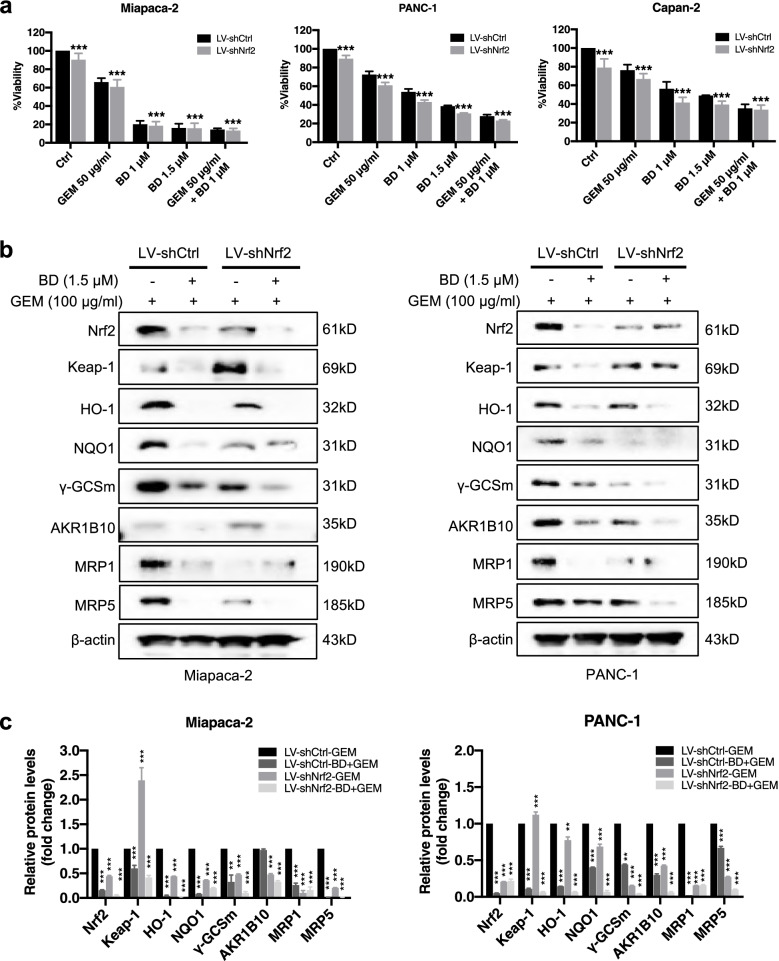
Knockdown of Nrf2 plus BD enhances the chemosensitivity to GEM in human PDAC cells. **a** Cell viability of the Nrf2-silenced and non-silenced Miapaca-2, PANC-1 and Capan-2 cells were measured by MTT assay. **b** Effects of Nrf2 knockdown on the protein levels of Nrf2, Keap1, HO-1, NQO1, γGCSm, AKR1B10, MRP1 and MRP5 in Miapaca-2, PANC-1 cells treated with BD and GEM. **c** The statistical analysis of relative protein level from Fig. 7b. Data were presented as the mean ± SD. *p < 0.05, **p < 0.01 and ***p < 0.001 compared with the control group

To further evaluate whether Nrf2 was involved in the BD-mediated sensitization of GEM in PDAC cells, we used Nrf2-depleted Miapaca-2, PANC-1 and Capan-2 cells to verify this hypothesis. As predicated, the knockdown of endogenous expression of Nrf2 led to significant sensitization of PDAC cells to cell death upon treatment with BD or GEM. On the other hand, co-treatment with BD and GEM exerted slight synergetic effects (Fig. 8a). To understand the potential mechanism underlying the enhanced antitumor effect of BD after Nrf2 knockdown, we assessed the protein level of Nrf2 using western blot. As shown in Fig. 8b-c, treatment with BD following silencing Nrf2 more significantly attenuated the expression of Nrf2 and its downstream protein than that in the cells without silenced Nrf2. Moreover, co-treatment with BD and GEM resulted in a more potent inhibitory effect. These results suggested that BD efficiently augmented the chemosensitivity of GEM by downregulating the Nrf2 expression, and the anti-PDAC effect was more potent when Nrf2 was depleted in the PDAC cells.

### BD suppresses the orthotopic PDAC tumor growth and sensitizes the efficacy to GEM by inhibiting Nrf2 pathway

**Fig. 9:**
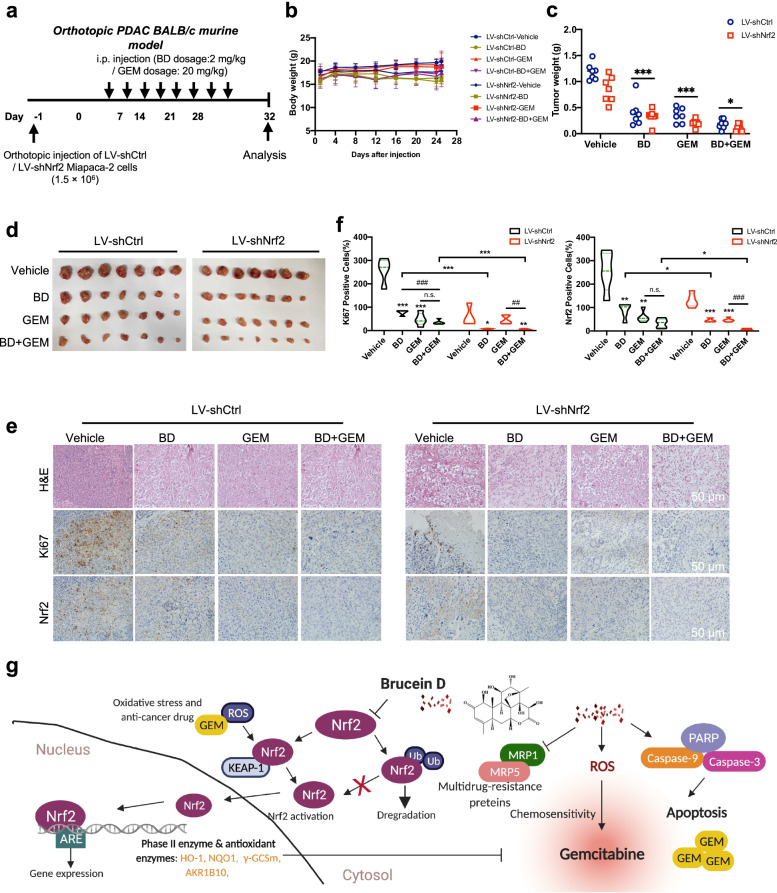
BD suppresses the orthotopic PDAC tumor growth and sensitizes the efficacy to GEM by inhibiting Nrf2 pathway. **a** Schematic presentation of the experiments on orthotopic PDAC murine model. **b** The body weight of mice was measured every 4 days after injection of cells. **c-d** Representative images of tumor and the tumor weight (*n* = 6–7). **e** Representative image of H&E and IHC staining of the resected tumors (Scale bar: 50 μm). **f** Statistical graphs of the IHC staining of Ki67 and Nrf2. **g** Schematic diagram of the regulation of Nrf2 signaling by BD treatment in PDAC cells. Data were presented as the mean ± SD. *p < 0.05, **p < 0.01 and ***p < 0.001 compared with the control group; n.s., no significant. ^##^*p* < 0.01 and ^###^*p* < 0.001 compared with the GEM group

To confirm the enhanced chemotherapeutic effect of BD in PDAC was Nrf2 dependent, we established the orthotopic mouse models using Miapaca-2 LV-shCtrl cells and Miapaca-2 LV-shNrf2 cells, respectively (Fig. 9a). No significant difference in the body weight was observed among different treatment groups (Fig. 9b). As depicted in Fig. 9c-d, Miapaca-2 LV-shNrf2 cells derived-orthotopic xenografts had a smaller tumor weight, as compared with the Miapaca-2 LV-shCtrl group following BD and GEM treatment, indicating that the co-treatment elicited a greater inhibitory effect. Analysis of excised tumors showed a similar reduction in cell density (H&E) in the Miapaca-2 LV-shNrf2 group compared with Miapaca-2 LV-shCtrl group. In addition, lower Nrf2 expression in the Miapaca-2 LV shNrf2-derived xenograft after BD treatment was confirmed by IHC staining. Furthermore, BD, GEM and their combination treatment also significantly attenuated the number of the Ki-67 positive cells in the LV-shNrf2 group when compared with that in the LV-shCtrl group (Fig. 9e-f). Taken together, our experimental results unambiguously indicate that BD exerted potent inhibitory effects on pancreatic tumor growth in the orthotopic PDAC mouse model, and the underlying mechanism involves the suppression of Nrf2 expression, thereby rendering the PDAC cells more sensitive to GEM.

## Discussion

Drug resistance continues to be one of the biggest challenges in cancer treatment, and exists across many types of cancer and chemotherapeutic regimens, including chemotherapy, targeted therapy and immunotherapy. Both intrinsic and acquired factors are involved in drug resistance. Overcoming drug resistance to improve chemosensitivity is a goal that has been pursued on many fronts, including basic science to uncover the underlying fundamental biological mechanisms and clinical trials testing new treatment strategies [2, 25–27]. Previous studies have reported that Nrf2 over-activity is a cardinal molecular mechanism of chemoresistance, and Nrf2 inhibition can reverse the drug resistance in many cancer types including PDAC [28–34]. Nrf2 also modulates a number of genes which control endogenous antioxidant protection and detoxification of ROS. The following genes including NQO1, HO-1, γ-GCSm, AKR1B10, MRP1 and MRP5 are known to be the important targets of Nrf2, and they are all involved in drug resistance in PDAC [35, 36]. Congruent with previous studies, we found that Nrf2 was highly expressed in tumor tissues of PDAC patients and predict poor prognosis, and silencing Nrf2 could markedly augment the sensitivity of GEM in PDAC. Our results unequivocally indicate that Nrf2 is a critical target for PDAC treatment, and inhibition of Nrf2 would therefore constitute an attractive strategy to sensitize PDAC cells to chemotherapeutic agents.

Several natural compounds have been shown to act as Nrf2 inhibitors and used as chemosensitizers in different types of cancer. Brusatol, a quassinoid originally isolated from *Bruceae Fructus*, a Chinese herbal medicine, potently reduces the protein level of Nrf2 in A549 cells, and sensitizes these cells to cisplatin and other chemotherapeutic drugs [37]. Digoxin was reported to sensitize GEM-resistant PDAC cells to GEM by inhibiting Nrf2 through suppressing the PI3K/Akt signaling pathway [38]. Wogonin, an O-methylated flavone isolated from *Scutellariae Radix*, was able to reduce the Nrf2 activity by suppressing the PI3K/Akt and Stat3/NF-κB signaling pathways and reverse chemoresistance [39, 40]. Our previous study revealed that BD could induce apoptosis in PDAC cells via modulating the activation of p38-mitogen activated protein kinase [21].

In the present study, we evaluated the anti-cancer effects of BD combined with GEM in PDAC cells. Our results revealed that BD could significantly improve the chemosensitivity of GEM. We also evaluated the therapeutic efficacy of the combination of BD and GEM in PDAC using KPC mice, a genetically engineered mouse model of PDAC widely used in PDAC research. Among which the *KRAS* gene encodes a protein that plays an essential role in cell signaling in normal tissue, through its activity as an ‘on/off’ switch for many signal transduction pathways, particularly those regulating cell division. Activation of mutations in pancreatic tumorigenesis causes *Kras* to be constitutively active, subsequently renders cells to grow and divide in an uncontrolled manner. p53, a tumor suppressor protein, stops cells from dividing too fast, and causes the damaged or mutated cells that might otherwise become tumorigenic to undergo apoptosis. It is well-known that p53 is often mutated in pancreatic tumors, meaning that mutated cells do not undergo apoptosis, thereby resulting in the occurrence of unregulated cell division. Because KPC mice carry mutations in genes which accurately mimics both the genetic and histologic changes of human PDAC, this model is therefore ideal for testing the efficacy of novel therapeutics [41, 42]. We found that BD could also enhance the chemosensitivity of GEM on KPC mice. Our findings revealed that treatment with combination of BD and GEM resulted in fewer cancer incidents, reduced number and size of macroscopic tumors, as compared with BD or GEM alone treatment. Biochemical and histological analyses showed that BD, GEM and their combination treatment did not affect the serum AST, ALT and creatinine levels and no obvious toxicity was observed in liver and kidney tissues of KPC mice, indicating that BD plus GEM treatment has good in vivo safety profile.

We subsequently tested BD for its ability to inhibit Nrf2 and to sensitize PDAC cells to apoptosis and PDAC tumors to GEM-based chemotherapy. As expected, BD exerted significant inhibitory effect on Nrf2 activity in a dose-dependent manner in PDAC cell lines. We also found that BD treatment did not alter Nrf2 mRNA level in PDAC cell lines but regulated and decreased Nrf2 protein levels by promoting its degradation. As proteasome activation contributes to anti-apoptotic effect of Nrf2 in tumor cells, not only the cellular response to anticancer drugs depends on phase II enzymes and detoxification genes expression, but also the responsiveness to death ligands would be affected by Nrf2 inhibition [43–45]. Accordingly, Nrf2-dependent resistance to anticancer drugs was significantly abrogated in all cell lines when pretreated with BD. Besides, the cellular ubiquitin level was increased after BD treatment and the BD-induced degradation of Nrf2 was effectively abolished by proteasome inhibitor MG-132. Our results amply indicate that the BD-induced Nrf2 downregulation involves the ubiquitin–proteasome-dependent pathway. Moreover, lentivirus depletion of Nrf2 together with BD treatment markedly sensitized anti-PDAC effect of GEM in both in vitro and in vivo pancreatic models.

Our present study has unambiguously demonstrated that BD, a naturally occurring quassinoid, is a potent inhibitor of Nrf2. Mechanistically, BD inhibits Nrf2 signaling via promoting degradation of Nrf2 protein and suppressing its downstream genes, thereby enhancing the chemosensitivity of GEM (Fig. 9g). Our experimental data strongly indicate that BD is worthy of being developed as a chemotherapeutic adjuvant for the treatment of PDAC, especially for those patients with aberrantly high Nrf2 expression.

## Conclusion

To conclude, our present study for the first time demonstrated that BD could enhance the chemosensitivity of GEM by modulating the transcription factor Nrf2. BD is a promising naturally occurring chemical worthy of further development into chemotherapeutic adjuvant to the GEM-based treatment for PDAC.

## Supplementary Information

**Additional file 1: Figure S1.** Nrf2 is overexpressed in PDAC and in GEM treated PDAC cells. **Figure S2.** Toxicological evaluation of BD combined with GEM in KPC mouse model. **Figure S3.** BD inhibits the Nrf2 activity. **Figure S4.** knockdown of Nrf2 enhances the chemosensitivity of GEM in PDAC. **Figure S5.** Activation of Nrf2 enhances the chemosensitivity of GEM in PDAC cells.

**Additional file 2.**

## Data Availability

All data generated or analyzed during the present study are included in this published article.

## Abbreviations

γ-GCSm: γ-Glutamylcysteine synthetase modifier subunit
AKR1B10: Aldo–keto-reductase
ALT: Alanine aminotransferase
AST: Aspartate aminotransferase
ARE: Antioxidant response element
ATCC: American Type Culture Collection
BCA: Bicinchoninic acid
BD: Brucein D
CHX: Cycloheximide
DMEM: Dulbecco’s Modified Eagle medium
DMSO: Dimethyl sulfoxide
FACS: Fluorescence-activated cell sorting
FBS: Fetal bovine serum
GEM: Gemcitabine
GEMM: Genetically engineered transgenic mice
GEPIA: Gene Expression Profiling Integrative Analysis
HO-1: Heme oxygenase-1
Keap1: Kelch-like ECH-associated protein 1
MAPK: Mitogen-activated protein kinase
MRP1: Multidrug-resistance-associated preotein-1
MRP5: Multidrug-resistance-associated preotein-5
MTT: 3-(4,5)-Dimethylthiahiazo(-2)-3,5-diphenytetrazoliumromide
NQO1: Quinone oxidoreductase
Nrf2: Nuclear factor-erythroid factor 2-related factor 2
PDAC: Pancreatic ductal adenocarcinoma cancer
PCR: Polymerase chain reaction
PFA: Paraformaldehyde
qRT-PCR: Quantitative real-time PCR
ROS: Reactive oxygen species
tBHQ: Tert-butylhydroquinone
