# Supplementary material for: Brucein D augments the chemosensitivity of gemcitabine in pancreatic cancer via inhibiting the Nrf2 pathway
Source: J Exp Clin Cancer Res. 2022 Mar 10;41:90. doi: 10.1186/s13046-022-02270-z (PMC8908700; doi:10.1186/s13046-022-02270-z)
Supplement: Supplementary file 1 — Additional file 1: Figure S1. Nrf2 is overexpressed in PDAC and in GEM treated PDAC cells. Figure S2. Toxicological evaluation of BD combined with GEM in KPC mouse model. Figure S3. BD inhibits the Nrf2 activity. Figure S4. knockdown of Nrf2 enhances the chemosensitivity of GEM in PDAC. Figure S5. Activation of Nrf2 enhances the chemosensitivity of GEM in PDAC cells. [file 13046_2022_2270_MOESM1_ESM.docx]

**Brucein D augments the chemosensitivity of gemcitabine in pancreatic cancer via inhibiting the Nrf2 pathway**

Juan Zhang^a^, Hongxi Xu^b^, William Chi Shing Cho^c^, Wah Cheuk^d^, Yang Li^b^, Qiong-Hui Huang^a^, Wen Yang^a^, Yan-Fang Xian^a,^*, Zhi-Xiu Lin^a,e,^*

**Methods**

**Chemicals and reagents**

Brucein D (BD, CAS: 21499-66-1) was isolated from *Bruceae Fructus* in our laboratory, and its structural identity was confirmed by comparing its NMR and HRMS data with those published previously [1]. Its purity was determined to exceed 98% by high-performance liquid chromatography analysis. Dimethyl sulfoxide (DMSO) and 3-(4,5)-dimethylthiahiazo (-2)-3,5-diphenytetrazoliumromide (MTT) were purchased from Sigma-Aldrich (St. Louis, MO, USA). Other chemicals and reagents used in the current investigation were of analytical grade.

**Quantitative real-time polymerase chain reaction (qRT-PCR)**

Total RNA isolation was extracted using TRIzol reagent (Thermo Fisher Scientific; MA, USA) following the manufacturer’s protocol. Reverse transcription PCR was performed using the PrimeScript RT Reagent (Takara, Bio, USA). The qRT-PCR analysis was performed in a Veriti Thermal Cycler (Applied Biosystems, Inc., FosterCity, CA, USA) using a SYBR Green Real Time PCR kit (Takara, Bio, USA). Data collection was performed using a StepOnePlus Real-Time PCR System Thermal Cycling Block (Applied Biosystems, Inc.). The primers for the qPCR reactions were as follows: Nrf2, 5’-TCCAGTCAGAAACCAGTGGAT-3’ and 5’-GAATGTCTGCGCCAAAAGCTG-3’; β-actin, 5’- GGACCTGACCTGCCGTCTAG-3’, 5’-GTAGCCCAGGATGCCCTTGA-3’. β-actin was used as an internal control. The PCR reaction conditions were 10 s at 95℃ followed by 40 cycles of 5 s at 95℃ and 20 s at 60℃.

**Cell viability assay**

Cells were plated into 96-well plates at 4,000 cells/well, and then treated with different concentrations of drugs for 24 or 48 h. Cell viability was determined by the MTT assay according to the manufacturer’s instruction as described in our previous study [2]. The absorbance was measured at 490 nm using a FLUOstar OPTIMA microplate reader (BMG Labtech, Offenbury, Germany). The half maximal inhibitory concentration (IC_50_) values were calculated from dose-response curves using GraphPad Prism 8 (La Jolla, California, USA).

**Colony formation assay**

Cells were seeded at specific cell densities in 6-well plates (500 cells/well), and the plates were incubated overnight. Subsequently, cells were treated with BD or GEM alone or combination for the indicated time. Drug-free growth medium was added and cells were further incubated for 14 days before being fixed with 4% formaldehyde and stained with 0.5% crystal violet. The number of colonies was counted under microscope.

**Supplementary Figures**

**
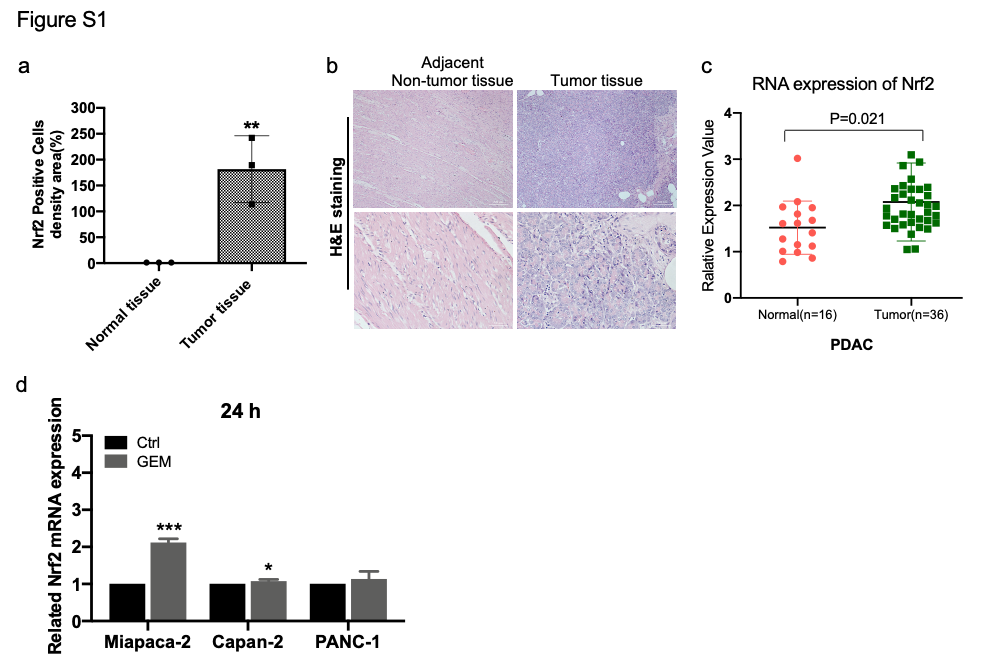
**

**Figure S1. Nrf2 is overexpressed in PDAC and in GEM treated PDAC cells** (a) Quantification of IHC for evaluation of Nrf2 expression. (b) The tumor and paired adjacent normal tissues in pancreatic cancer patients were stained with HE (Scale bar: 50 & 200 μm). (c) Analysis of Nrf2 mRNA expression in normal pancreas (n = 16) and pancreatic carcinoma tissues (n = 36) by the Oncomine database. (d) The mRNA expression of Nrf2 in Miapaca-2, Capan-2, and PANC-1 cells. The cells were treated with GEM for 24. Data were presented as the mean ± SD. *p < 0.05, **p < 0.01 and ***p < 0.001 compared with the control group.


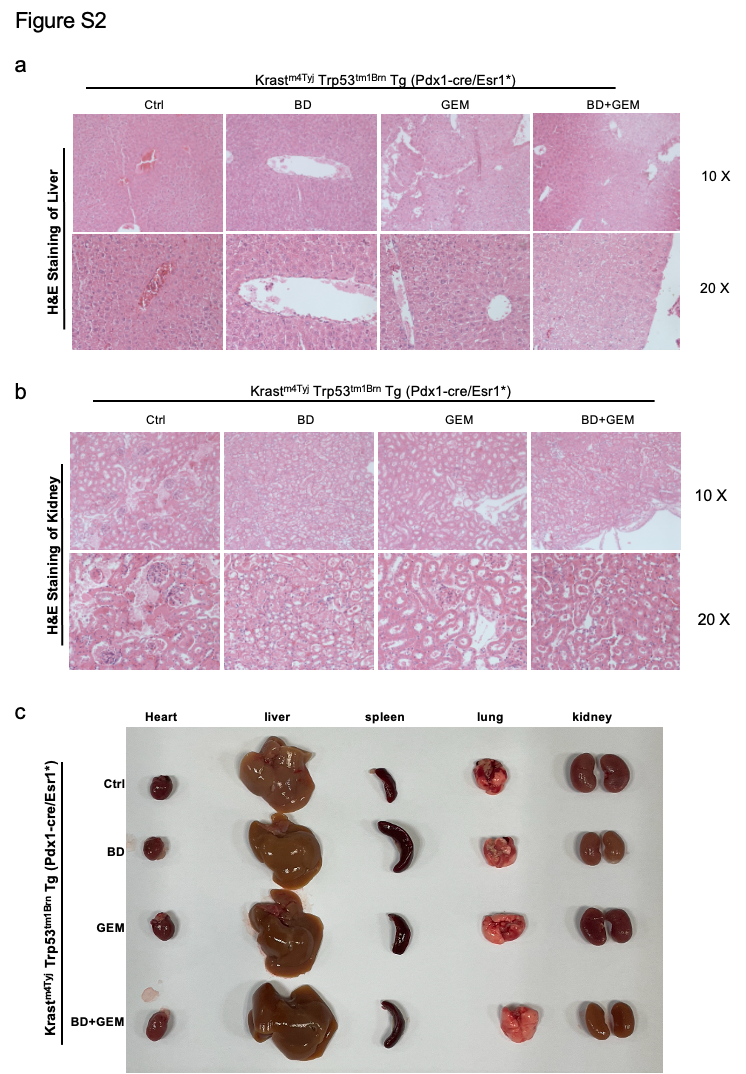


**Figure S2.** Toxicological evaluation of BD combined with GEM in KPC mouse model. (a-b) Representative image of pathological morphology of liver and kidney in KPC mice were evaluated by H&E staining. (c) Image of organelle morphology of heart, liver, spleen, lung, and kidney treated with BD, GEM and their combination on 62th day observation.


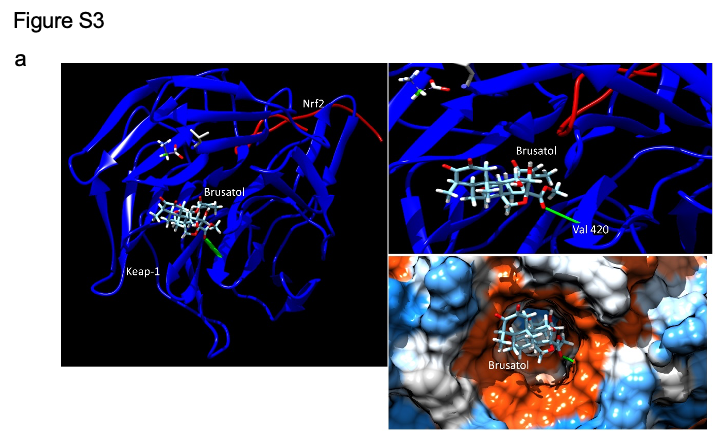


**Figure S3. BD inhibits the Nrf2 activity.** (a) Nrf2 inhibitor Brusatol (BR) docking with Nrf2. The blue ribbon represents the Keap-1 amino acid chain, the red ribbon represents the Nrf2 amino acid, the green bonds represent the hydrogen bond. Val is the abbreviation of valine.


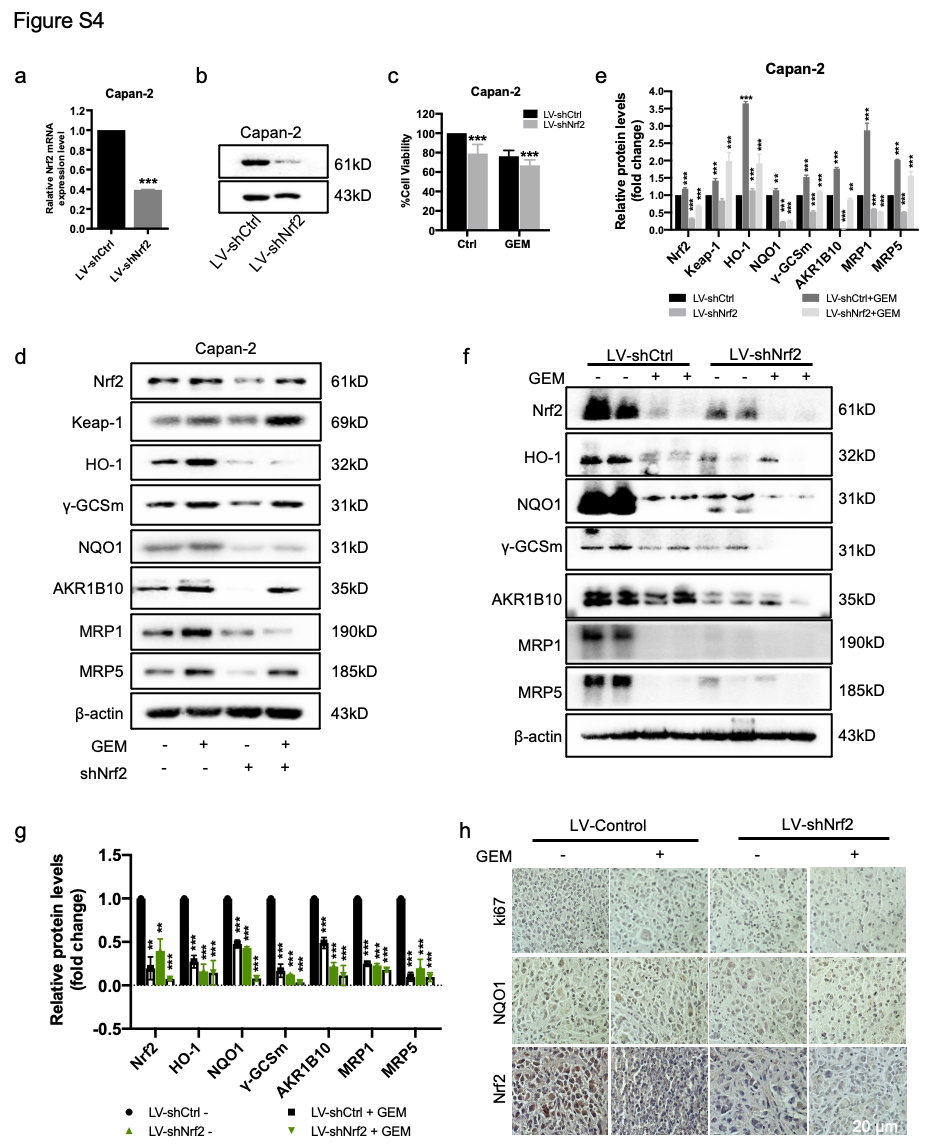


**Figure S4. knockdown of Nrf2 enhances the chemosensitivity of GEM in PDAC.** (a-b) The Nrf2 mRNA and protein were stably knockdown in Capan-2 cells. LV-shNrf2, recombinant lentivirus deletion Nrf2; LV-shCtrl, recombinant lentivirus negative control. (c) Effects of GEM treatment on the viability in the Capan-2 Nrf2-silenced and non-silenced cells. (e-d) Effects of GEM treatment on the protein levels of Nrf2, Keap1, HO-1, NQO1, γGCSm, AKR1B10, MRP1 and MRP5 in the Nrf2-knockdown Capan-2 cells. (f-g) Effects of GEM on the protein levels of Nrf2, HO-1, NQO1, AKR1B10, γGCSm and MRP1 in tumor tissues. (h) Paraffin-embedded orthotopic tumor tissues were sectioned and stained with IHC for evaluation of Ki-67, Nrf2 and NQO1 expression (Scale bar: 20 μm). Data were presented as the mean ± SD. **p* < 0.05, ***p* < 0.01 and ****p* < 0.001 compared with the control group.


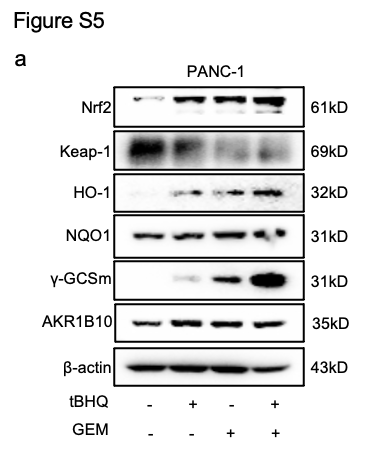


**Figure S5. Activation of Nrf2 enhances the chemosensitivity of GEM in PDAC cells.** (a) Effects of Nrf2 activation on protein levels of HO-1, NQO1, γGCSm and AKR1B10 in PANC-1 cells following tBHQ, GEM or their combination treatment.

Reference:

1. Ren D, Villeneuve NF, Jiang T, Wu T, Lau A, Toppin HA, et al. Brusatol enhances the efficacy of chemotherapy by inhibiting the Nrf2-mediated defense mechanism. Proc Natl Acad Sci U S A. 2011;108(4):1433-8.

2. Xian YF, Lin ZX, Mao QQ, Ip SP, Su ZR, Lai XP. Protective effect of isorhynchophylline against beta-amyloid-induced neurotoxicity in PC12 cells. Cell Mol Neurobiol. 2012;32(3):353-60.
