# Supplementary material for: Brucein D augments the chemosensitivity of gemcitabine in pancreatic cancer via inhibiting the Nrf2 pathway
Source: J Exp Clin Cancer Res. 2022 Mar 10;41:90. doi: 10.1186/s13046-022-02270-z (PMC8908700; doi:10.1186/s13046-022-02270-z)
Supplement: Supplementary file 2 — Additional file 2. [file 13046_2022_2270_MOESM2_ESM.pdf]

Additional file 1: Table S1: **List of primary antibodies used in this study.** Abbreviations: CST: purchased from the Cell Signalling Technology, Danvers, MA, USA; SCB: Santa Cruz Biotechnology, Santa Cruz, CA, USA.

Primary antibody	Obtained from	Cat. No	Dilution
Nrf2	SCB	sc-365949	1:500
Nrf2	Proteintech	16396-1-AP	1:100 (IHC)
Keap-1	CST	8047S	1:1500
HO-1	SCB	sc-136960	1:500
NQO-1	SCB	sc-32793	1:1000
γ-GCSm	SCB	sc-22754	1:1000
AKR1B10	Proteintech	18252-1-AP	1:1000
MRP1	SCB	sc-18835	1:500
MRP5	SCB	sc-376965	1:500
β-actin	SCB	sc-69879	1:1000
GAPDH	Abcam	ab-181602	1:1000
ubiquitin	SCB	sc-166553	1:1000
Caspase-3	SCB	sc-7272	1:1000
Caspase-9	SCB	sc-56076	1:1000
PARP	SCB	sc-7150	1:1000

Additional file 1: Table S2: Primer sequences used for the genotyping of transgenic mice.

Genes	Primer Sequences
Kras^{tm4Tyj}	22907 (F): 5’-TGTCTTTCCCCAGCACAGT-3’<br>22908 (Common): 5’- CTGCATAGTACGCTATACCCTGT-3’<br>oIMR9592 (R): 5’-GCAGGTCGAGGGACCTAATA-3’
Trp53^{tm1Brn}	oIMR8543 (F): 5’-GGTTAAACCCAGCTTGACCA-3’<br>oIMR8544 (R): 5’-GGAGGCAGAGACAGTTGGAG-3’
Pdx1-Cre	21557 (F): 5’-GCAGTGGAGAACTGTCAAAGC -3’<br>oIMR7338 (F): 5’-CTAGGCCACAGAATTGAAAGATCT-3’<br>oIMR7339 (R): 5’-GTAGGTGGAAATTCTAGCATCATCC-3’<br>oIMR9377 (R): 5’- ATGTTTAGCTGGCCCAAATG-3’
